# Association of Aldosterone Synthase Polymorphism (*CYP11B2* -344T>C) and Genetic Ancestry with Atrial Fibrillation and Serum Aldosterone in African Americans with Heart Failure

**DOI:** 10.1371/journal.pone.0071268

**Published:** 2013-07-30

**Authors:** Adam Bress, Jin Han, Shitalben R. Patel, Ankit A. Desai, Ibrahim Mansour, Vicki Groo, Kristin Progar, Ebony Shah, Thomas D. Stamos, Coady Wing, Joe G. N. Garcia, Rick Kittles, Larisa H. Cavallari

**Affiliations:** 1 Department of Pharmacy Practice, University of Illinois at Chicago, Chicago, Illinois, United States of America; 2 Department of Medicine, Section of Cardiology, University of Illinois at Chicago, Chicago, Illinois, United States of America; 3 Department of Medicine, Institute for Personalized Respiratory Medicine, University of Illinois at Chicago, Chicago, Illinois, United States of America; 4 Department of Medicine, Section of Hematology/Oncology, University of Illinois at Chicago, Chicago, Illinois, United States of America; 5 Division of Health Policy and Administration, University of Illinois at Chicago, Chicago, Illinois, United States of America; 6 Department of Medicine, Section of Pulmonary, Critical Care, Sleep and Allergy, University of Illinois at Chicago, Chicago, Illinois, United States of America; Howard University, United States of America

## Abstract

The objective of this study was to examine the extent to which aldosterone synthase genotype (*CYP11B2*) and genetic ancestry correlate with atrial fibrillation (AF) and serum aldosterone in African Americans with heart failure. Clinical data, echocardiographic measurements, and a genetic sample for determination of *CYP11B2* -344T>C (rs1799998) genotype and genetic ancestry were collected from 194 self-reported African Americans with chronic, ambulatory heart failure. Genetic ancestry was determined using 105 autosomal ancestry informative markers. In a sub-set of patients (n = 126), serum was also collected for determination of circulating aldosterone. The *CYP11B2* −344C allele frequency was 18% among the study population, and 19% of patients had AF. Multiple logistic regression revealed that the *CYP11B2* −344CC genotype was a significant independent predictor of AF (OR 12.7, 95% CI 1.60–98.4, p = 0.0150, empirical p = 0.011) while holding multiple clinical factors, left atrial size, and percent European ancestry constant. Serum aldosterone was significantly higher among patients with AF (p = 0.036), whereas increased West African ancestry was inversely correlated with serum aldosterone (r = −0.19, p = 0.037). The *CYP11B2* −344CC genotype was also overrepresented among patients with extreme aldosterone elevation (≥90th percentile, p = 0.0145). In this cohort of African Americans with chronic ambulatory heart failure, the *CYP11B2* −344T>C genotype was a significant independent predictor of AF while holding clinical, echocardiographic predictors, and genetic ancestry constant. In addition, increased West African ancestry was associated with decreased serum aldosterone levels, potentially providing an explanation for the lower risk for AF observed among African Americans.

## Introduction

Heart failure is a common and costly phenotype, with apparent differences in prevalence between African Americans and non-African Americans [1], [2]. African Americans display differences in heart failure pathogenesis, drug response, and treatment outcomes compared to other ethnic groups, with significant genetic contribution [3], [4], [5], [6], [7]. African Americans also carry a paradoxically low risk for atrial fibrillation (AF), despite having a higher prevalence of well-established AF risk factors such as heart failure, hypertension, diabetes and larger body size [8], [9], [10], [11], [12]. Interestingly, higher European ancestry among African Americans was predictive of incident AF in a previous study [13]. However, African Americans remain under-represented in genetic studies in heart failure, particularly as they relate to risk of AF development.

Activation of the renin-angiotensin-aldosterone system (RAAS) plays a critical role in the pathogenesis of both heart failure and AF [14], [15], [16], [17]. Specifically, aldosterone binding to the mineralocorticoid receptor stimulates myocyte apoptosis, leading to cardiac fibrosis, which is central to both heart failure and AF pathophysiology [18], [19]. Mineralocorticoid receptors are up-regulated in atrial myocytes in the setting of AF, potentially amplifying the effects of aldosterone binding [20]. As further evidence of the importance of aldosterone and mineralocorticoid receptors in the pathogenesis of AF, mineralocorticoid receptor antagonists (MRAs) have been shown to reduce the risk of new-onset AF in predominantly European populations with systolic heart failure [21], [22], [23].

Aldosterone synthase is the enzyme that catalyzes the final reaction to generate aldosterone. The aldosterone synthase gene (*CYP11B2)* consists of 9 exons and is localized to chromosome 8q22 [24]. A common SNP in the promoter region of the *CYP11B2* gene, c. −344T>C (rs1799998) occurs in approximately 30% of African Americans and 46% of Europeans [25], with 4-times greater affinity for the steroidogenic transcription factor 1 (SF-1) and increased aldosterone excretion reported with the −344C allele [26], [27]. In clinical studies, the −344C allele has been linked to increased left ventricular size in Europeans and both systemic hypertension and ischemic stroke in Asians [28], [29]. The −344CC genotype was also predictive of risk for AF in an Israeli population with heart failure and predictive of both worsening left ventricular remodeling and increased risk of death and hospitalization in African Americans with heart failure [30], [31]. In contrast, a study of 67 African Americans with chronic systolic HF linked the −344C allele to lesser cardiac remodeling [32].

Despite the central role of aldosterone in heart failure progression and risk for AF in heart failure, the relationship between the *CYP11B2* −344C allele and AF in African Americans remains unknown. We, therefore, evaluated the association between the −344T>C SNP and AF in an ambulatory, African American, chronic heart failure population. Given the potential role for genetic heterogeneity and ancestry in heart failure outcomes, genetic ancestry was also analyzed.

## Methods

### Ethics statement

The study was approved by the Institutional Review Board at the University of Illinois at Chicago. Written, informed consent was obtained from all patients prior to study enrollment.

### Study population

African Americans (by self-report) at least18 years of age, with a diagnosis of heart failure (either with reduced or preserved ejection fraction) for at least 3 months were included. Additional inclusion criteria were treatment with an ACE inhibitor or angiotensin receptor blocker (or if contraindicated, the combination of hydralazine and nitrates) for at least 6 months with no change in doses of these medications for at least 2 months. Patients with a history of liver disease were excluded.

### Study procedures

After obtaining written, informed consent, a buccal cell or venous blood sample was collected for determination of genotype and, in a subset of patients, additional blood was collected for determination of serum aldosterone concentration. Since serum aldosterone exhibits diurnal variation and may be influenced by body position, all blood samples were drawn between 8 am and 1 pm while patients were seated after being upright for at least 2 hours. [33] Samples were stored at −80°C until further analysis. Demographic, clinical, and social data were collected at the time of enrollment. AF was defined by a University of Illinois Hospital and Health Science System (UI-Health) cardiologist at study enrollment and diagnosed by documentation in either the electronic medical record or evidence on 12-lead electrocardiography (ECG) and/or Holter monitoring, as previously published [34]. Atrial fibrillation cases associated with a recent surgery or hyperthyroidism were excluded.

### Echocardiography

Transthoracic echocardiographic studies were performed within 12 months of enrollment using an Acuson SC2000^TM^ ultrasound system. Echocardiographic measurements of left ventricular end diastolic diameter (LVEDD) were performed using standard 2D and M-Mode methods. Left ventricular ejection fraction was assessed using 2D methods and the Simpson method of discs, and left atrial size was determined using linear measurements as outlined by the American Society of Echocardiography [35]. Severity of mitral regurgitation was determined using color Doppler and the PISA method (when appropriate) as outlined by the American Society of Echocardiography [36].

### Aldosterone assay

Samples for aldosterone were assayed using a commercially available kit containing I-125-labeled aldosterone (Beckman Coulter, Brea, CA), as previously described [37]. All samples were assayed in duplicate. Intra-assay and inter-assay coefficients of variation for this assay were 1.5% and 1.9% respectively [37].

### Genotyping

Genomic DNA was isolated from buccal cells or whole blood using a Puregene^®^ kit (Qiagen, Valencia, CA). Genotyping for the *CYP11B2* -344T>C (rs1799998) polymorphism was done via PCR and capillary sequencing, with primers and annealing temperatures shown in Table S1. Genotype results were verified using a different primer set. Each genotype was scored by two independent investigators blinded to AF status. Individual genetic ancestry was determined for each person using 105 autosomal DNA ancestry informative markers for West African, Native American, and European genetic ancestry using published methods [38], [39]. Each participant was then scored from 0% to 100% for individual estimates of West African, Native American and European ancestry.

### Data analysis

Creatinine clearance was calculated using the equation of Cockcroft and Gault and ideal body weight [40]. Hardy-Weinberg equilibrium was tested by χ2 analysis. Normally distributed continuous data are presented as mean ± SD and were compared by unpaired t-tests and analysis of variance. Continuous data that were non-normally distributed are presented as median (IQR) and were compared with Mann Whitney U and Kruskal Wallis tests. The χ^2^ or Fischer's exact test was used to compare categorical data, and the Cochran-Armitage trend test was used to compare allele frequencies between groups. Multiple logistic regression permitted tests of association (odds ratio) between *CYP11B2* -344T>C and presence of AF while holding clinical factors, echocardiographic measurements, and genetic ancestry constant. Dominant, additive, recessive and genotypic effects models were all used to test the association of *CYP11B2* −344T>C genotype and presence of AF. Based on previous data, risk factors for AF included as covariates in the multiple logistic regression models were age, sex, body size, systemic hypertension, diabetes, coronary artery disease, creatinine clearance, left atrial size, mitral regurgitation and genetic ancestry [41], [42], [43], [44], [45], [46]. Marginal standardization was used for the final logistic regression model to estimate adjusted prevalence differences between genotype groups [47]. Bootstrapping was used to quantify the confidence intervals of the prevalence difference generated from the marginal standardization [48]. Given the low prevalence of the CC homozygous genotype, we used permutation to generate a distribution genotypic effects under the assumption of a true null hypothesis, which creates an empirical p value for the association of the *CYP11B2* −344T>C recessive effects model and AF [49].

For the exploratory analysis of serum aldosterone, linear regression (ordinary least squares) was used to examine the association between genetic ancestry and aldosterone levels. Serum aldosterone was natural–log transformed to produce a more normal distribution of regression residuals, as done previously [50]. Mean log serum aldosterone was compared between genotype and AF groups by the unpaired t-test. We also examined the association between genotype and extreme elevation of log aldosterone despite standard heart failure therapy, which was defined as a serum log aldosterone level at or above the 90^th^ percentile for the study population, using Fisher's exact test.

A two-sided p value of less than 0.05 was considered as statistical significance. Statistical analyses were performed with the SAS software package, version 9.2 (SAS Institute, Cary, NC, USA), and Stata/SE software, Version 12.1 (StataCorp, College Station, TX, USA).

## Results

A total of 194 African Americans were enrolled and successfully genotyped. Table 1 shows the clinical characteristics, echocardiographic measurements, and genetic ancestry of the entire study cohort and by *CYP11B2* −344T>C genotype. Patients were well treated, with nearly all receiving an ACE inhibitor or angiotensin II receptor antagonist and 97% taking a β-blocker. All 20 patients taking an MRA had the −344 CT or TT genotype. Clinical and echocardiographic characteristics were similar between *CYP11B2* −344T>C genotype groups, with the exception of AF, which was more prevalent with the −344CC versus TC or TT genotype (71 versus 17%, p = 0.003), and creatinine clearance, which was lower with the −344CC genotype (45±13 versus 65±26 mL/min, p = 0.04).

**Table 1:** Characteristics of the total cohort and according to *CYP11B2* −344T>C genotype.

Characteristic	Total Cohort (n = 194)	TT (n = 131 )	TC (n = 56)	CC (n = 7)
Age (yrs)	55±14	56±14	51±15	60±7
Male sex	93 (48)	71 (54)	18 (32)	4 (57)
BMI, kg/m^2^	34±11	34±11	34±9.9	35±14
NYHA Class	2.5 (2–3)	2.5 (2–3)	3 (2–3)	2 (1–3)
Ischemic Etiology	44 (23)	32 (24)	11 (20)	1 (14)
Diabetes	63 (32)	47 (36)	13 (23)	3 (43)
Systemic Hypertension	160 (82)	110 (84)	44 (79)	6 (86)
Atrial Fibrillation	37 (19)	22 (17)	10 (18)	5 (71)*
CrCl (mL/min)	65±26	63±25	71±29	45±13**
B-Blocker use	188 (97)	126 (96)	56 (100)	6 (86)
ACE inhibitor or ARB use	192 (99)	130 (99)	55 (98)	7 (100)
MRA use	20 (10)	17 (13)	3 (5)	0
Loop Diuretic Use	112 (58)	76 (58)	30 (53)	6 (86)
Thiazide Diuretic Use	7 (4)	5 (4)	2 (4)	0
Digoxin Use	59 (30)	37 (28)	18 (32)	4 (57)
Echocardiograph measurements				
LA Size (mm)	44±8	44±7.9	43±8.1	47±13
LVEDD (cm)	5.9±1.0	5.9±0.9	5.8±1.1	5.7±1.4
Ejection Fraction (%)	30±14	30±14.1	28±14	35±17
Mod-Severe or Severe MR	32 (16)	20 (15)	12 (21)	0
Ejection Fraction				
<40%	145 (75)	96 (73)	45 (80)	4 (57)
40–50%	32 (16)	26 (20)	4 (7)	2 (29)
>50%	17 (9)	9 (7)	7 (13)	1 (14)
Genetic ancestry (%)				
European	18±11	17±11	18±12	22±12
West African	75±13	76±13	74±14	70±13
Native American	7±6	7±5	8±6	8±6

Count (%), mean ± SD, or median (interquartile range).

* p = 0.007 value for comparison between genotype groups.

** p = 0.03 value for comparison between genotype groups.

ACE, angiotensin converting enzyme; AF, atrial fibrillation; ARB, angiotensin receptor blocker; BMI, body mass index; CrCl, creatinine clearance; MR, mitral regurgitation; MRA, mineralocorticoid receptor antagonist; LA, left atrium; LVEDD, left ventricular end diastolic diameter; NYHA, New York Heart Association.

Table 2 displays the clinical characteristics, echocardiographic measurements, and genetic ancestry by AF status. AF was present in 37 (19%) participants. As expected based on past reports [1], [13], [17], [41], patients with AF were older, had worse renal function and larger left atrial size, and were more likely to have moderately-severe or severe mitral regurgitation compared to those without AF.

**Table 2:** Patient characteristics according atrial fibrillation status.

Characteristic	AF (n = 37)	No AF (n = 157)	p Value
Age (yrs)	61±13	54±14	0.007
Male sex	21 (57)	72 (46)	0.23
BMI, kg/m^2^	34±11	34±11	0.92
NYHA Class	2 (1–3)	3 (2–3)	0.06
Ischemic Etiology	6 (16)	38 (24)	0.29
Diabetes	9 (24)	54 (34)	0.23
Systemic Hypertension	32 (86)	128 (82)	0.47
CrCl (mL/min)	50±22	68±26	<0.001
B-Blocker	36 (97)	152 (97)	0.99
ACE inhibitor or ARB	37 (100)	155 (99)	0.99
MRA Use	3 (8)	17 (11)	0.76
Loop Diuretic Use	21 (57)	91 (58)	0.99
Thiazide Diuretic Use	1 (3)	6 (4)	0.99
Digoxin Use	11 (30)	48 (31)	0.92
Echocardiographic Measurements			
LA Size (mm)	50±7.6	43±7.7	<0.001
LVEDD (cm)	5.9±1.1	5.9±1.0	0.93
Ejection Fraction (%)	30±12	30±15	0.97
Mod-Sev or Severe MR	10 (27)	22 (14)	0.055
Genetic ancestry (%)			
European	18±12	18±11	0.77
West African	74±13	75±13	0.65
Native American	8±6	7±5	0.65

Count. (%), mean ± SD, or median (interquartile range).

ACE, angiotensin converting enzyme; AF, atrial fibrillation; ARB, angiotensin receptor blocker; BMI, body mass index; CrCl, creatinine clearance; MR, mitral regurgitation; MRA, mineralocorticoid receptor antagonist; LA, left atrium; LVEDD, left ventricular end diastolic diameter; NYHA, New York Heart Association.

Table 3 shows the genotype and allele frequencies in the total cohort and by AF status. Genotype distribution did not deviate from Hardy-Weinberg equilibrium (χ2 expected versus observed, p = 0.74). The −344C allele frequency in the total cohort was consistent with previous reports in Africans and African Americans and was significantly higher in those with AF [31].

**Table 3:** *CYP11B2* −344T>C genotype and allele frequencies.

Genotype or Allele frequency	Total Cohort (n = 194)	AF (n = 37)	No AF (n = 157)	P value
Genotype frequency, n (%)				
TT	131 (68)	22 (59)	109 (69)	0.002*
TC	56 (29)	10 (27)	46 (29)	
CC	7 (3.6)	5 (14)	2 (1.3)	
Allele Frequency, n (%)				
T	318 (82)	54 (73)	264 (84)	0.014**
C	70 (18)	20 (27)	50 (16)	

* by χ^2^analysis for difference between genotype groups and presence of AF.

** by Cochran-Armitage trend test for differences of allele frequencies between AF groups.

**Figure 1:**
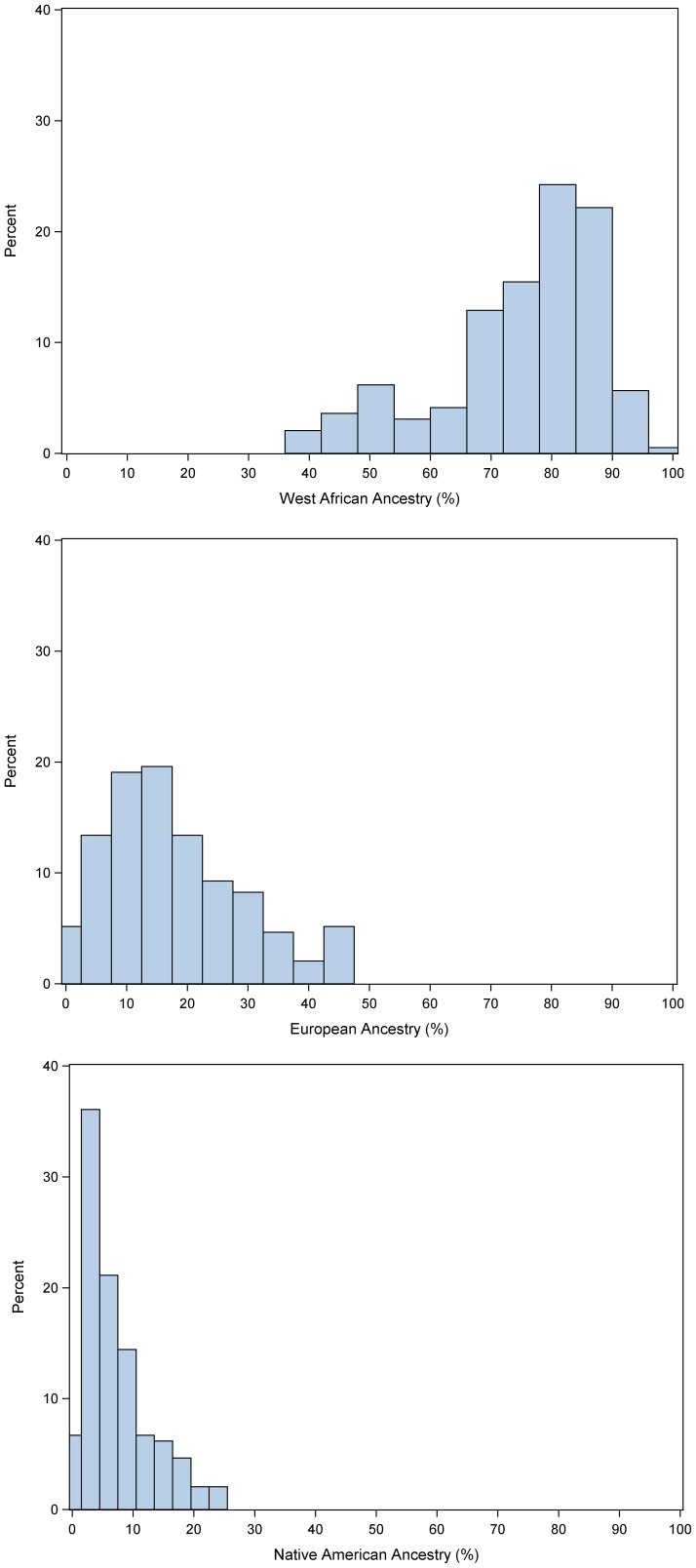
Percent of patients of West African, European, and Native American ancestry as determined by ancestral informative markers, in self-identified African Americans.

Figure 1 displays the distribution of genetic ancestry. On average, the cohort had 75% West African, 18% European and 7% Native American ancestry. Ancestry was not associated with AF by either bivariate or multivariate analyses.

### *CYP11B2* −344 T>C and atrial fibrillation

Table 4 shows the results of the multiple logistic regression models testing the association of the *CYP11B2* −344 T>C polymorphism with AF while adjusting for clinical and echocardiographic covariates and genetic ancestry. Covariates were selected for inclusion in the model based on previous data [41], [42], [43], [44], [45], [46] and included age, sex, body size (BMI), creatinine clearance, systemic hypertension, diabetes, coronary artery disease, left atrial size, mitral regurgitation and European ancestry. Including percent European ancestry allowed for control for population admixture and also for potential confounding as European ancestry was previously associated with AF in African Americans [51]. We specified the *CYP11B2* −344 T>C genotype four different ways: Model 1, allelic effects (0, 1, 2 for number of C alleles carried); Model 2, genotypic effects (indicator variables for CC, CT and TT as the reference group); Model 3, recessive effects (0 for CT or TT, 1 for CC); and Model 4, dominant effects (0 for CC or CT, 1 for TT). Both Models 1 and 3 demonstrated an association between the *CYP11B2* −344T>C genotype and AF, with the greatest effects observed with the −344CC genotype. Also in Model 1, each 5 millimeter increase in left atrial size was associated with a 69% increase in the odds of AF. The size of association between left atrial size and AF was similar between genotype model specifications. Creatinine clearance was associated with AF in all models, with each 10 ml/min increase in clearance decreasing odds of AF by approximately 25%. None of the other clinical and echocardiographic covariates were significant (p<0.05) predictors of AF.

**Table 4:** Predictors of atrial fibrillation in multiple logistic regression analysis.

Variable	Adjusted OR	95% CI	P value	P_emp_ value*
Model 1 (Allelic Effects Model)				
* CYP11B2* −344C, (0,1,2)	2.14	1.05–4.32	0.035	
LA Size (per 5mm)	1.65	1.21–2.25	0.001	
CrCl (per 10 ml/min increase)	0.74	0.56–0.96	0.024	
Model 2 (Genotypic Effects Model)				
* CYP11B2* −344 CC	13.4	1.70–105	0.014	
* CYP11B2* −344 CT	1.24	0.47–3.3	0.662	
LA Size (per 5mm)	1.68	1.23–2.31	0.001	
CrCl (per 10 ml/min increase)	0.75	0.57–0.99	0.040	
Model 3 (Recessive Effects Model)				
* CYP11B2* −344 CC	12.7	1.60–98.4	0.015	0.011
LA Size (per 5mm)	1.69	1.23–2.31	0.001	
CrCl (per 10 ml/min increase)	0.75	0.57–0.99	0.041	
Model 4 (Dominant Effects Model)				
* CYP11B2* −344 CC or CT	1.867	0.77–4.52	0.166	
LA Size (per 5mm)	1.643	1.21–2.23	0.001	
CrCl (per 10 ml/min increase)	0.726	0.56–0.95	0.018	

LA, left atrial; CrCl, creatinine clearance.

Adjusted for age, sex, body size (BMI), mitral regurgitation, systemic hypertension, coronary artery disease, diabetes mellitus, left atrial size, creatinine clearance, and percent European ancestry.

* Empirical p value generated by permuting the CC genotype term in the logistic model (10,000 reps). The p values represents the proportion of permutations that led to a coefficient on the CC term at least as large as the one observed in the actual sample.

### Marginal standardization and adjusted prevalence difference estimates

**Figure 2:**
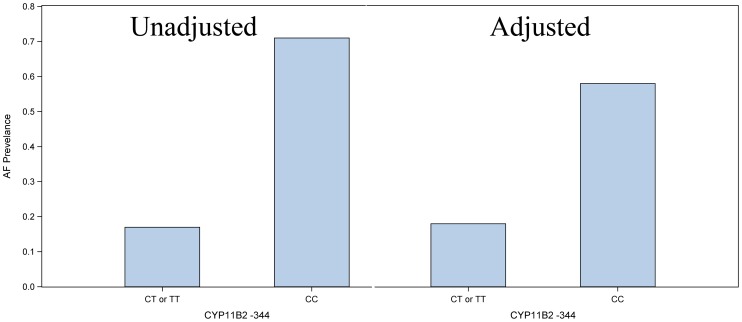
Unadjusted and adjusted (marginal standardization) proportions of individuals among *CYP11B2*−344T>C genotype groups with AF.

We used the recessive effects model, Model 3, to generate adjusted prevalence differences using marginal standardization. Figure 2, shows the unadjusted association of *CYP11B2* −344CC genotype with AF status (prevalence difference of 0.54, 95% CI 0.20 to 0.88, p = 0.003) on the left. Figure 2 on the right displays marginal standardization, which is the average prevalence difference between genotype groups adjusted for age, sex, body size, creatinine clearance, systemic hypertension, diabetes, coronary artery disease, left atrial size, mitral regurgitation, and European ancestry. The *CYP11B2* CC genotype was associated with an adjusted AF prevalence difference of 40 percentage points (95% CI 9 to 67).

To enhance the strength of our association estimate for Model 3, we generated empirical p values using permutation. This test assumes that the null hypothesis is true (no association), and shows the probability of obtaining our observed association estimate simply by chance. The p value on our observed coefficient for *CYP11B2* −344 CC genotype was 0.011 for the recessive effects model. This corresponds to the proportion of permutations that led to a coefficient at least as large as the one observed in the actual sample. This suggests it is unlikely that the association observed in our data is simply by chance.

### *CYP11B2* association with aldosterone levels

**Figure 3:**
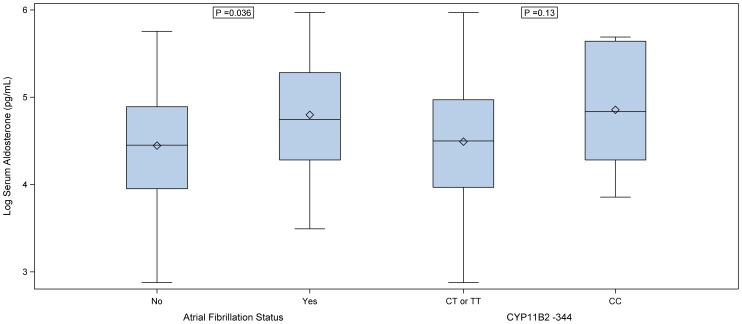
Serum aldosterone level by AF status and *CYP11B2* genotype.

To explore the possible causal pathway underlying the *CYP11B2* association with AF, we measured serum aldosterone in a subset of 126 patients who provided samples for this analysis. None of these patients were on an MRA. The median (range) aldosterone concentration in the study population was 90 (18 to 392) pg/ml. Thirteen patients (10%) had aldosterone levels in the upper 90th percentile (>196 pg/ml). Figure 3 shows the distribution of log serum aldosterone by *CYP11B2* genotype and AF status. Log aldosterone concentration was significantly higher in those with versus without AF (p = 0.036). There was no significant association between log aldosterone concentration examined as a continuous variable and *CYP11B2* −344T>C genotype (p = 0.13). However, 50% of patients with the *CYP11B2* −344CC genotype versus 8% with the TC or TT genotype had log aldosterone levels in the upper 90th percentile for the study sample (OR 11, 95% CI 2.0 to 62, p = 0.015).

### Genetic ancestry and aldosterone levels

**Figure 4:**
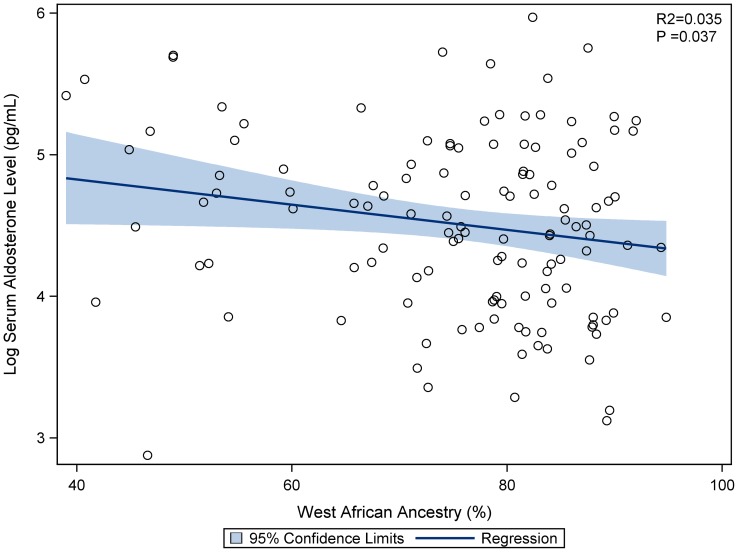
Association between West African ancestry and aldosterone levels.

Figure 4 displays the correlation between West African ancestry and log serum aldosterone levels in 126 subjects. In our cohort of self−reported African Americans, West African ancestry was associated with lower log serum aldosterone levels (R2 = 0.035, p = 0.037), while European ancestry was associated with higher levels (R2 =  0.031, p = 0.048). There was no association between Native American ancestry and serum aldosterone (p = 0.30).

## Discussion

Our study has two main findings. First, the *CYP11B2* −344T>C genotype was associated with a significant increase in odds of AF in an African American, ambulatory heart failure population, after controlling for a rich set of clinical and echocardiographic covariates as well as controlling for population admixture using genetic ancestry. This observation is consistent with previous findings in Asian and Middle Eastern populations [30], [52]. Specifically, the *CYP11B2* −344CC genotype was associated with a 2.4-fold increase in the odds of AF among an Israeli cohort with systolic HF [30]. Similarly, a meta-analysis of six studies including over 2,700 Asian patients with essential hypertension, hypertensive heart disease, or heart failure showed a 2-fold increase in odds of AF with the CC genotype [52]. Consistent with previous studies, our data also support a recessive effects model for the *CYP11B2* −344T>C genotype link to AF [30]. While the odds ratio from the recessive effects model in our study is greater in size than that previously reported, this might be reflective of lower precision due to smaller sample size in the current study, or alternatively, could reflect differences in the patient populations under study.

Consistent with previous studies of patients with hypertension or dilated cardiomyopathy, we also found that the *CYP11B2* −344CC genotype was over-represented among individuals with extreme elevation of aldosterone, defined as the 90th percentile for the study population [53], [54]. This finding sheds light on the mechanism potentially explaining increased risk for AF with the *CYP11B2*−344CC genotype. Further, evidence that the *CYP11B2* −344CC genotype is associated with extremes in serum aldosterone levels may help to explain the previously observed associations between the −344CC genotype and greater left ventricular mass and decreased event-free survival among African Americans with heart failure [29], [31], [53]. Specifically, higher aldosterone levels with the −344CC genotype could lead to greater cardiac fibrosis and remodeling, which are important contributors to heart failure-related morbidity, mortality and risk for developing AF [19], [55].

Our second major finding from our study is the novel association between genetic ancestry and serum aldosterone levels. Specifically, we found that among African Americans with heart failure, greater European ancestry was correlated with higher serum aldosterone concentrations, and greater West African ancestry was correlated with lower aldosterone levels. These finding are consistent with previous observations of lower plasma aldosterone in African Americans compared to Europeans with various stages of hypertension [56], [57]. Moreover, the link between European ancestry and elevated aldosterone may provide insight into the mechanism underlying the association between European ancestry and increased risk of AF in African Americans in the Atherosclerosis Risk in Communities (ARIC) Study [51]. In particular, our data support the hypothesis that the genetic ancestry effect on incident AF could be due, in part, to differences in aldosterone levels by ancestry. However, we did not observe a significant association between ancestry and AF in our study, which is in contrast to data from the ARIC study.[51] It is possible that our study was underpowered to detect such an association, especially with the modest effect size (HR 1.17) observed in ARIC between European ancestry and incident AF [51].

The implication of our findings that *CYP11B2* genotype is associated with both AF risk *and* elevated aldosterone is that *CYP11B2* may be a useful biomarker to identify heart failure patients at risk for AF in whom aldosterone antagonism may attenuate such risk. In addition to increasing the risk for stroke, AF can also exacerbate heart failure symptoms and reduce exercise capacity [58], [59]. As such, the ability to predict patients at greater risk for AF, in whom modalities could be instituted to ameliorate this risk, could potentially lessen heart failure-related morbidity. There is strong evidence that aldosterone antagonism with an MRA in systolic heart failure improves survival and reduces heart failure -related morbidity, including AF [21], [22], [23]. There are also data that the MRA spironolactone attenuates the deleterious effects of aldosterone in AF at the level of atrial tissue, where mineralocorticoid receptors appear to be up-regulated, lending support to the idea that an MRA may attenuate the risk for AF in heart failure patients with a −344CC genotype [20]. However, the role of MRAs in heart failure patients with the *CYP11B2* −344 CC genotype-associated AF risk has yet to be ascertained.

It is important to note that there have been discrepant results with the *CYP11B2* −344T>C variant and its association with aldosterone secretion and the presence of cardiovascular disease [26]. Along with type-I error due to sample size, one of several explanations is confounding association due to population admixture, which we have addressed by adjusting for genetic ancestry [60]. Allele and genotype frequencies in our study are similar to those from the African American Heart Failure Trial (A-HeFT) [31]. However, there are significant differences in *CYP11B2* −344T>C allele and genotype frequencies among ethnic groups. African Americans have a lower frequency of the CC genotype (∼3–10%) than both Europeans (∼20–30%) and Asians (∼7–12%), which may explain the intra-ancestral discrepancy in certain association studies [25], [30], [31], [52], [61]. Some of the previous studies focused on patient populations with cardiovascular diseases other than heart failure, and failed to find an association between −344CC genotype and AF [61], [62]. It is possible that aldosterone regulation plays a greater role in the pathogenesis of AF in heart failure patients than patients with other diseases considering the significant aldosterone involvement in the ventricular remodeling and cardiac fibrosis [14], [16], [19].

There are several limitations to our study. First, the incidence of AF is complex, multi-factorial, and subject to many confounders. Although we controlled for a rich set of important clinical and echocardiographic covariates in our analysis, other potential variables such as alcohol consumption and family history were not explored. Similar to other pharmacogenetic studies, this study comprised a small sample size limiting the precision of association estimates. In addition, the −344CC genotype occurs at low frequency in African Americans, and thus, our association estimates with this genotype are exploratory and require confirmation. However, the permutation analysis of the recessive effects model suggests that these findings are unlikely to be simply due to chance (p value = 0.019). Further, this study population was of African descent, and thus, caution is warranted in drawing conclusions for other racial groups. Finally, contribution by variants not interrogated in this study is also possible, as there may be other, unobserved, polymorphisms in or near *CYP11B2* that may be functionally important [34], [63], [64].

## Conclusion

In summary, in a cohort of African Americans with chronic ambulatory heart failure, the *CYP11B2* −344CC genotype was a significant and independent predictor of AF beyond conventional clinical and echocardiographic predictors of AF and genetic ancestry. The *CYP11B2* −344CC genotype was also associated with extreme elevation of serum aldosterone, providing insight into the mechanism underlying AF risk with the *CYP11B2* genotype. Also, among self-reported African Americans, increasing West African ancestry was associated with decreased serum aldosterone levels. Whether strategies, such as aldosterone antagonism with an MRA, might impact the risk for AF conferred by the −344CC genotype remains to be determined.

## Supporting Information

Table S1 PCR primers.
(DOCX)
