# Supplementary material for: Association of Aldosterone Synthase Polymorphism (CYP11B2 -344T>C) and Genetic Ancestry with Atrial Fibrillation and Serum Aldosterone in African Americans with Heart Failure
Source: PLoS One. 2013 Jul 30;8(7):e71268. doi: 10.1371/journal.pone.0071268 (PMC3728110; doi:10.1371/journal.pone.0071268)
Supplement: Table S1 — PCR primers. (DOCX) [file pone.0071268.s001.docx]

Table S1. PCR primers

| SNP | Primer sequence (5’-3’) | Annealing temp |
| --- | --- | --- |
| *CYP11B2* -344T>C  *CYP11B*2 -344T>C (For verification) | F: GTACGTGGACATTTTCTGCAGTTT  R: ATGGGGACTTTATCTTATCGTGAG  F: GAGACCCCATGTGACTCCAG  R: GTCCATGCTGGTGGAAGGT | 60°C  60°C |
